# The Importance of Person Centered Coaching on Physical Activity in Older Adults

**DOI:** 10.1093/geroni/igaa057.3384

**Published:** 2020-12-16

**Authors:** Lenny Chiang-Hanisko, Sue Graves, Jean Joseph-Rendel, David Newman, Debroah D’Avolio

**Affiliations:** Florida Atlantic University, Boca Raton, Florida, United States

## Abstract

Osteoarthritis (OA) is the most common form of arthritis and imposes a substantial social and economic burden on American society. Evidence suggests that physical exercise provides health-related benefits to reduced osteoarthritis pain, stress and depression. However, exercise alone may not be sufficient to make necessary lifestyle changes to reduce OA pain. This study aimed to determine the effectiveness of Person Centered Coaching (PCC) and Health Education (HE) on physical activity of exercising older adults with OA pain. 14 exercising older adults were included in this experimental, randomized, pre and posttest study. Age ranged from 69 -88 years (mean = 77) with most participants identifing as non-Hispanic whites, all currently retired and 71% female. Participants completed OA Diary, 3 PROMIS short-form instruments (Pain Interference, Perceived Stress and Instrumental Support) and Personal Growth Initiative Scale. Data was collected over six weeks to include baseline and post-intervention measurements using repeated measures analysis. The result indicated the PCC treatment group had lower scores on pain interference and perceived stress compared to the HE group. The PCC group also had increased higher level on personal growth and instrumental support compared to the HE group. The results suggest that additional interventions are needed to enhance exercise effectiveness in reducing pain, inflammation and stress. The intervention (PCC), has the potential to provide a positive impact on OA outcomes for exercising older adults by increasing self-knowledge and self-monitoring of OA pain, as well as facilitating persistence with exercise.

